# From Bench to Bureaucracy: The Path from Academic Research to Science Policy

**DOI:** 10.1089/dna.2021.0673

**Published:** 2022-01-12

**Authors:** Rushika C. Wirasinha

**Affiliations:** Department of Health, Australian Government, Canberra, Australia.

**Keywords:** science policy, health, careers

## Abstract

As the COVID-19 pandemic shines a spotlight on the importance of science to support evidence-based policy, Rushika Wirasinha, PhD writes of a career path available to academic researchers–science policy.

## Introduction

Science, and the profound impact it has on society, has never held such a captive audience as right now. Scientific discoveries are providing us with the answers to fight the COVID-19 pandemic and the rapid dissemination of scientific findings through mainstream and social media has contributed to society becoming more engaged in science. From uncovering the molecular details of the SARS-CoV-2 virus, the development of COVID-19 vaccines, the implementation of various COVID-19 public health measures, to monitoring the impacts of those measures, the responsibility of scientists and policy makers is under the microscope now more than ever.

Our society will only be able to navigate this pandemic if rigorous academic research is translated into effective evidence-based policy. To achieve this, academic researchers need to engage with policy makers and clearly outline their scientifically informed view. Alternately, there is an opportunity for academic and scientific training to be effectively used in becoming a policy expert. Perhaps current events are highlighting a different and important career path—science policy.

## The Traditional Academic Career Path Is Not for Everyone

Embarking on a career in science is exciting. Being the first person to uncover a novel finding and make an important contribution to knowledge building provides a sense of accomplishment and marks you as part of the academic research community.

The traditional career path in academia consists of the following (not necessarily linear) steps: (1) complete a PhD, (2) build a publication record, (3) apply for grant funding, (4) initiate strategic collaborations, (5) establish an independent research group, and (6) gain recognition as a world-leading expert to build your research empire. However, many eager early career researchers face competition and challenges at each of these steps.

For the past decade, the number of PhD completions in Australia has more than doubled, further increasing the competition for the very limited number of academic research roles (Universities Australia, 2020). Some of the challenges faced by Australian early career researchers have been widely observed and documented, and include job insecurity, an unsupportive workplace culture, and inadequate mentorship (Christian *et al.*, 2021).

## From Academic Research to Science Policy

While undertaking my PhD and postdoctoral research, I saw many exceptional colleagues encounter difficulties in pursuit of academic success. As is the case for many early career researchers, I had doubts about the sustainability of a future in academia and seeking information on alternate career paths was challenging (Alechine, 2019). It was also difficult to initiate a conversation with peers and supervisors for fear of betraying the system and the people who had supported me and invested much time and energy to help me make strides down the competitive academic career path.

Eventually, I ventured into science-based health policy at the Australian Government Department of Health. After leaving the academic bubble and now working in policy, I see the value that rigorous scientific training adds to both public and private sectors and, by extension, the community more broadly. This value must be acknowledged and promoted within academia—there is a need to broaden perceptions of possible career paths available to academically trained researchers, including in science-based policy.

As COVID-19 has exemplified, the contributions of academic research to policy making have had a measurable impact on public health responses. For example, using data modeling (informed by knowledge of SARS-CoV-2 biology) to implement COVID-19 public health measures. One avenue is for academic researchers and medical experts to directly provide advice to policy makers. This advice may be based on firsthand evidence generated directly from their research, or knowledge gained through their expert review of literature in their field of knowledge. It is necessary to effectively communicate scientifically complex concepts in a way that is accessible to policy makers.

Another more direct avenue is for academic scientists to themselves become trained in policy, and to become embedded as policy makers actively contributing knowledge and critical thinking skills to decision-making processes. A career in science policy is often described as an “alternate” career path for academic researchers. But from my experience, to embed scientific thinking in policy making requires academically minded individuals who are inquisitive, analytical, and strategic in their thinking, as well as driven and keen to contribute to society.

## Science Policy as a Career Path

Although not essential to make the career transition, science policy programs provide on-the-job training for those with no prior policy experience. The longest running program is the Science and Technology Policy Fellowships (STPF) established by the American Academy for the Advancement of Science (AAAS) in 1973 (https://www.aaas.org). Through this program, >3400 PhD-level scientists and engineers have gained firsthand experience in developing policies across government, nonprofits, and industry to address challenges facing society.

The Australian Science Policy Fellowship program commenced in 2018 as a 3-year pilot program, and following its success, is now a permanent program in the public service (www.chiefscientist.gov.au). The program aims to model the AAAS STPF program, providing a pathway for early to mid-career scientists to become embedded in federal government departments to fast track their development of skills and experience in public policy. Administered by the Office of the Chief Scientist of Australia, the Fellowship program provides additional training, mentoring, and alumni networking opportunities.

During my fellowship year, I was placed within the Australian Government Department of Health. After an initial adjustment to learn new acronyms and writing styles, I came to appreciate and utilize my skill set formed in academia. Similar to research, policy work requires strategic thinking, program management, synthesis of complex information, data analysis, writing articles and reports, engaging with diverse stakeholders, working collaboratively and communicating effectively. It was fulfilling to work on challenging issues as a team and to propose innovative solutions. New experiences and challenges allowed me to see how the unique academic skill set added value to conversations with colleagues, influenced outcomes, and improved government policy making.

I now have a greater appreciation of the multitude of skills that PhD-trained scientists can offer beyond subject-matter expertise. Academic researchers should have confidence in the value of their skills and how they can be translated and applied in environments outside of academia.

In the short time I have been involved in policy development, I have observed that the key to effective evidence-informed policy is to (1) identify the gaps, (2) analyze and synthesize scientific knowledge, (3) accurately communicate the information in a format suitable to the audience, and (iv) define proposed actions that may be translated into policy initiatives.

In academia, a scientist may rely on evidence (e.g., hypothesis-driven research, statistically significant data, and replicability) to support an argument. For science policy, that evidence must be coupled with an approach that is responsive to the needs and perspectives of the broader public and as well as government priorities. Where the goals of the scientist and policy maker intersect, policies are more effectively developed and implemented. An ability to understand the divergent (and convergent) viewpoints of academia, the general public, and government is a valuable perspective.

## Future Perspectives and Conclusions

There are many exciting and varied opportunities to make valuable scientific contributions in fields beyond traditional academic research. Through a career pivot from immunology to science-based health policy, I have experienced firsthand the fulfilment and growth such a career change can bring. I am involved in frontline discussions on emerging scientific discoveries and play a role in translating these into actions and outcomes for the community. I can see the community-level implications of my science policy work.

There has never been a more important time to capitalize on the importance of scientific thinking in policy decision making. Scientists have a key role to support government established policies important to health and livelihoods, and to effectively communicate these decisions to the broader public.
